# Comparing diagnostic prostate cancer biomarkers using a framework of clinically meaningful criteria

**DOI:** 10.1002/bco2.70275

**Published:** 2026-09-22

**Authors:** Shu Wang, Behnam Nabavizadeh, Ashwin Ramaswamy, Meena Davuluri, Tenny R. Zhang, Michael Tzeng, Nathan Feiertag, Jim C. Hu

**Affiliations:** ^1^ Department of Urology New York‐Presbyterian Hospital/Weill Cornell Medical Center New York New York USA; ^2^ The Milton and Carroll Petrie Department of Urology Icahn School of Medicine at Mount Sinai New York New York USA; ^3^ Albert Einstein College of Medicine Bronx New York USA

**Keywords:** biomarkers, diagnosis, prostate cancer

## Abstract

**Objectives:**

To synthesize existing biomarker literature and generate a framework for meaningful comparison between five commonly used diagnostic prostate cancer biomarkers—prostate health index (*phi*), 4KScore, SelectMDx, ExoDx and MyProstate Score (MPS)—to provide evidence of greatest practical value to providers managing men with an elevated prostate‐specific antigen (PSA) who are considering prostate biopsy.

**Materials and methods:**

Ten biomarker characteristics reflecting *validity*, *usability* and *utility* were designated as most clinically salient in guiding diagnostic prostate cancer biomarker selection. Whereas *usability* reflects how well a biomarker individualizes risk and optimizes patient concerns, *utility* reflects a biomarker's clinical usefulness. A systematic review of the biomarker literature from 2010 to 2022 was performed, and the criteria for each biomarker characteristic were first inductively defined and subsequently determined for each respective biomarker.

**Results:**

Nearly all biomarkers possess wide *validity*, with *phi*, 4KScore and MPS validated in Black populations. Compared to other biomarkers, 4KScore had the best *usability*. For urine‐based biomarkers, the better *usability* of ExoDx versus MPS is offset by its worse *validity* and *utility*. All serum‐based biomarkers have very high discriminative accuracy and were consequently more accurate in detecting high‐grade prostate cancer versus urine‐based biomarkers. Overall, 4KScore had the best *utility*.

**Conclusion:**

Our framework enables meaningful comparison of diagnostic prostate cancer biomarkers using criteria most salient to patients and clinicians. We demonstrate that the 4KScore currently possesses the best overall features; despite emerging evidence, more clinical utility, comparative and non‐White population validation studies are needed.

## 1 INTRODUCTION

In the last 10 years, the use of novel diagnostic prostate cancer biomarkers as an adjunctive test to serum prostate‐specific antigen (PSA) has become increasingly routine. These biomarkers aim to address current limitations posed by screening with PSA alone: PSA has a lower specificity when compared to many other common screening tests for malignancy^1^; screening is associated with unneeded biopsies, overdiagnosis and overtreatment^2^; PSA values are not easily interpretable because a continuum of risk exists at all levels of PSA.^3^ However, widespread utilization of such highly accurate novel diagnostic biomarkers is complicated by a problem of too much choice—currently, providers can choose from *seven* biomarkers without much comparative evidence on which may be best for their patient.

Thorough examination of the prostate cancer biomarker literature yields a number of reviews that meticulously describe the research on different biomarkers.^4^, ^5^, ^6^ While helpful, these exhaustive and dense presentations are not practically useful because study details—for instance, a validation study of ExoDx had 519 patients and found an area under the curve (AUC) of 0.73^7^—are of questionable relevance to the practising urologist in comparison to practical concerns, such as whether ExoDx incorporates other clinical information (e.g., prior negative biopsy) or whether it has been validated for use in non‐White men. The current literature does not conveniently facilitate provider biomarker selection for a given patient.

Therefore, the aim of this review was to synthesize the literature to generate a framework for meaningful comparison between diagnostic prostate cancer biomarkers and provide information to address practical concerns and questions of providers. Specifically, we evaluated biomarkers which inform a decision regarding initial prostate biopsy, namely, the prostate health index (*phi*), 4KScore, SelectMDx, ExoDx and MyProstate Score (MPS). We hope to emphasize the most clinically salient biomarker comparisons to facilitate the most informed choice for men considering prostate biopsy.

## 2 MATERIAL AND METHODS

Ten characteristics reflecting the *validity*, *usability* and *utility* of diagnostic prostate cancer biomarkers were designated as most salient to help guide diagnostic prostate cancer biomarker selection and inform decision making on whether to pursue prostate biopsy (Table 1). These characteristics were deductively determined based on reasonable assumptions of what a *good* diagnostic prostate cancer biomarker should accomplish; by contrast, the characteristics' accompanying criteria were inductively determined based on available evidence presented in the biomarker literature.

**TABLE 1:** Ten clinically salient characteristics for diagnostic prostate cancer biomarkers.

#	Characteristic	Evaluation	Criteria
	*Validity*		
C1	Wide external validation	Yes	≥1 validation study with ≥2000 patients OR ≥50 HGPC detected in three validation studies
		No	∅
C2	Validation in non‐White populations	Black/Asian/Latino	≥1 validation study with ≥100 Black, Asian, and/or Latino patients, respectively
		Broad	Validated in ≥2 non‐White populations (e.g., Black and Asian)
		None	∅
	*Usability*		
C3	Incorporation of an individual's clinical data into score	Yes	Incorporates individual's clinical data (e.g., positive DRE, prior negative biopsy) into score
		No	∅
C4	Reported as an individual's probability of disease	Yes	Reports an individual's probability of disease (e.g., ‘your risk of prostate cancer is 8%’)
		No	∅
C5	Non‐invasive sampling type	Yes	Measured via serum or urine without requiring DRE
		No	Requires ‘prostate massage’ and/or prostate volume calculation via transrectal ultrasound
C6	Intended clinical use	Broad	PSA > 10 ng/mL *and* prior negative biopsy
		Narrow	Either PSA > 10 ng/mL *or* prior negative biopsy
		Restrictive	Only in biopsy‐naïve men with a PSA of 2–10 ng/mL
	*Utility*		
C7	High‐grade measured as clinical endpoint	Yes	Specifically validated to discriminate for HGPC
		No	∅
C8	Discriminative accuracy for high‐grade disease	Very high	AUC for HGPC ≥0.8
		High	… 0.75–0.799
		Medium	… 0.7–0.749
		Low	…<0.7
C9	Diagnostic utility in clinical practice	Likely beneficial	Decreased prostate biopsy rates and LGPC detection *without* an undue reduction in HGPC detection
		Possibly beneficial^a^	Decreased prostate biopsy rates^a^
		Likely harmful	Undue reduction in HGPC detection
		Possibly harmful^a^	Increased prostate biopsy rates^a^
		Unclear	Clinical utility study did not meet above criteria and/or clear confounders prevent interpretation
		Not done	∅
C10	Oncologic endpoints in clinical practice	Yes	≥1 study where biomarker improved discrimination of metastasis or disease‐specific survival
		No	∅

Abbreviations: AUC, area under the curve; DRE, digital rectal exam; HGPC, high‐grade prostate cancer; LGPC, low‐grade prostate cancer; PSA, prostate‐specific antigen.

^a^ No historic/prospective control prevents determination of the change in LGPC/HGPC incidence.

Biomarker *validity* reflects the extent to which the biomarker has been studied in a large number of patients. Characteristics most salient to biomarker *validity* include (**C1**) wide external validation and (**C2**) validation in non‐White populations. Biomarker *usability* reflects the extent to which the biomarker individualizes predictive modelling of disease risk and optimizes patient concerns in clinical practice. Characteristics most salient to biomarker *usability* include (**C3**) incorporation of individual clinical data into score, (**C4**) reporting individual probability of disease, (**C5**) non‐invasive sampling type and (**C6**) intended clinical use. Biomarker *utility* assesses the extent to which a biomarker is clinically useful and performs as intended by improving discrimination of high‐grade prostate cancer with the end goal of preventing metastasis and cancer‐specific mortality. Characteristics most salient to biomarker *utility* include (**C7**) high‐grade measured as clinical endpoint, (**C8**) discriminative accuracy for high‐grade prostate cancer (quantified as AUC, describing the ability of a test to diagnose patients with or without the disease in question), (**C9**) diagnostic utility in clinical practice and (**C10**) oncologic endpoints in clinical practice.

We performed a systematic literature search (Table S1) on PubMed, Scopus and Web of Science of *phi*, 4KScore, SelectMDx, ExoDx and MPS from January 2010 to September 2022 following Preferred Reporting Items for a Systematic Review and Meta‐analysis of Diagnostic Test Accuracy methodology.^6^ Two other diagnostic biomarkers—ConfirmMDx and PCA3—were excluded from analysis are they are currently indicated only men with a prior negative prostate biopsy. Additional references cited in articles were cross‐screened to identify articles not included in our initial search. We excluded letters, editorials, correspondence and articles in which the primary language was not English.

Notably, our methodology was such that not all relevant papers need to be included. While characteristics (**C3**), (**C4**), (**C5**), (**C6**) and (**C7**) describe the biomarker itself, characteristics (**C1**), (**C2**), (**C8**), (**C9**) and (**C10**) illustrate how the biomarker has been studied in validation cohorts and utilized in clinical practice. For these latter characteristics, articles with the highest level of evidence were selected among those meeting specific criteria for any given characteristic. For example, a biomarker was considered to possess wide external validation (**C1**) if *one* study included a validation cohort of ≥2000 patients; alternatively, if one validation study had 2000 patients and another had 3000 patients, the latter was selected all other things being equal. The full evaluative methodology of each characteristic's criteria is detailed in Appendix S1.

After completing our evaluation, we obtained feedback from recognized experts of the given biomarkers for our overall framework of biomarker characteristics and their accompanying criteria, as well as our assessment of each biomarker in case we had missed or misunderstood any of the literature. Feedback from those experts (recognized in the Acknowledgements) was then incorporated into our final evaluation.

## 3 RESULTS

The results for the 10 salient biomarker characteristics for *phi*, 4KScore, SelectMDx, ExoDx and MPS are shown in Table 2.

**TABLE 2:** Assessment of *phi*, 4KScore, SelectMDx, ExoDx and MPS.

#	Characteristic	PHI	4K	SelectMDx	ExoDx	MPS
	*Validity*					
C1	Wide external validation	Yes	Yes	Yes	No*	Yes
C2	Validation in non‐White populations	Broad	Black*	None*	None*	Black
	*Usability*					
C3	Incorporation of an individual's clinical data into score	No	Yes	Yes	No	No
C4	Reported as an individual's probability of disease	No	Yes	No	No	Yes
C5	Non‐invasive sampling type	Yes	Yes	No	Yes	No
C6	Intended clinical use	Broad	Broad	Restrictive*	Broad	Restrictive*
	*Utility*					
C7	High‐grade measured as clinical endpoint	No*	Yes	Yes	Yes	Yes
C8	Discriminative accuracy for high‐grade disease	Very high	Very high	Very high	Medium*	High*
C9	Diagnostic utility in clinical practice	Unclear*	Possibly beneficial*	Not done	Unclear*	Not done*
C10	Oncologic endpoints in clinical practice	Yes	Yes	No	No	No

* Indicates assessments open to reasonable disagreement, discussed in Results, Section 3, and Appendix S1, Section S2.

We report several notable findings. First, while there is significant evidence for biomarker *validity*, few studies evaluated biomarker *utility* (e.g., diagnostic utility, oncologic endpoints). For example, our assessment that *phi* has (**C9**) *unclear* diagnostic utility was made based on conflicting findings across just three clinical utility studies. Second, with the exception of ExoDx, all biomarkers possess (**C1**) wide external *validity*; *phi*, 4KScore and MPS were also well validated in Black populations, whereas little data on non‐White populations were available for SelectMDx and ExoDx (**C2**). Notably, *phi* has the broadest validation in non‐White, non‐Black populations as demonstrated in studies with majority Hispanic, Middle Eastern and Asian cohorts. Third, among all biomarkers, 4KScore has the best overall *usability* as a test that: (**C3**) incorporates clinical data into the score, (**C4**) reports an individual's probability of disease; (**C5**) is non‐invasive; and (**C6**) has broad intended clinical use. While SelectMDx and MPS individualize predictive modelling of disease risk to a degree, neither is non‐invasive nor has broad intended clinical use. In contrast, while *phi* and ExoDx are non‐invasive and have broad intended clinical use, both are otherwise agnostic to other personalized predictors of high‐grade disease. Lastly, for *utility*, all serum‐based biomarkers (*phi*, 4KScore, SelectMDx) had (**C8**) *very high* discriminative accuracy. Consequently, serum‐based biomarkers were more accurate in the detection of high‐grade prostate cancer than urine‐based biomarkers (i.e., ExoDx, MPS). In this context, 4KScore has the best overall *utility* as a test that assesses (**C7**) high‐grade disease as a clinical endpoint, (**C8**) has a very high discriminative accuracy for high‐grade disease, (**C9**) has possible beneficial diagnostic utility in clinical practice and (**C10**) has oncologic endpoints in clinical practice.

In the succeeding section, we will review a subset of assessments open to reasonable disagreement (indicated with an asterisk in Table 2). An in‐depth assessment of all biomarker characteristics for *phi*, 4KScore, SelectMDx, ExoDx and MPS is located in Appendix S2.

### 3.1 Prostate Health Index

The *phi* predicts probability of having any prostate cancer; it does not report high‐grade prostate cancer as a clinical endpoint (**C7**) to providers ordering the test. For *phi*, characteristic **(C9)** ‘Diagnostic utility in clinical practice’ was determined as *Unclear*. Three studies have evaluated the diagnostic utility of *phi* in real clinical practice. White et al. reported on the clinical utility of *phi* in 506 men across four large urology practices compared to historical controls. In this study, use of *phi* resulted in a significant reduction in biopsies performed (36% *phi* vs. 60% historic) as well as an overall reduction in the detection of low‐grade prostate cancer (10% vs. 18%).^8^ Notably, all men received the *phi* test; use was not based on provider discretion. However, further analysis by Ehdaie et al. in a correspondence letter noted one high‐grade cancer was missed for about every three biopsies not performed.^9^ Moreover, 39 patients initially planned for biopsy were switched to surveillance despite receiving an ‘elevated risk’ *phi* score of 36–55+; this may indicate challenges in understanding and implementing *phi*'s risk thresholds in clinical practice. In their response, White et al. presented additional data demonstrating a 7% decrease in high‐grade prostate cancer detection from 18.3% to 11.3% in the historic and prospective cohorts, respectively, in contrast to an 8.5% decrease in GG1 detection. By contrast, a comparable single‐institution study that relied on provider discretion rather than a fixed *phi* threshold found a 9% reduction in prostate biopsies driven by decreased detection of negative and low‐grade (GG1) disease, without a corresponding decrease in high‐grade (GG2+) cancer detection.^10^ This discrepancy may reflect that White et al.'s ≥36 cut‐off for ‘elevated risk’ exceeds the ~27 threshold supported by other validation and population‐screening studies as an appropriate balance of sensitivity and specificity for high‐grade disease, rather than reflecting an inherent limitation of *phi*.^11^, ^12^ Incorporation of *phi* into nomograms alongside multiparametric MRI has since been shown to further improve detection of clinically significant disease while reducing biopsies.^13^ Given these conflicting findings—and given that the same absence of a historic control did not preclude a *possibly beneficial* (**C9**) rating for 4KScore—the diagnostic utility of *phi* in clinical practice was determined to be *Unclear* rather than likely harmful.

### 3.2 4Kscore

For the 4KScore, characteristics **(C2)** ‘Validation in non‐White populations’ and **(C9)** ‘Diagnostic utility in clinical practice’ deserve explanation. The 4KScore was validated in Black men **(C2)** in a large prospective study at eight Veterans Affairs medical centres in which the majority of the intended use population identified as African American (205 men). While one retrospective, population‐based study of Latino and Asian men in the multiethnic cohort demonstrated high discriminative accuracy (AUC 0.77 [0.75–0.81]) for the detection of aggressive prostate cancer, this study did not meet our criteria for a validation study as not all participants were biopsied, high‐grade prostate cancer was not used as a primary outcome and the authors were unable to identify any men with Gleason 7 disease due to unforeseen data limitations.^14^, ^15^

Although a clinical utility study was performed evaluating the 4KScore demonstrating a 65% decrease in biopsies, the absence of a historic or prospective control group prevents interpretation of the resultant change in incidence of low‐grade and high‐grade prostate cancer.^16^ However, because authors reported the distribution of 4KScore results in non‐biopsied patients some inference on cancer outcomes is possible, as the 4KScore score itself defines the probability of high‐grade prostate cancer. Therefore, the diagnostic utility of this biomarker in clinical practice was considered *possibly beneficial* (**C9**).

### 3.3 SelectMDx

For SelectMDx, there are currently no validation studies in non‐White populations (**C2**). SelectMDx has only been validated in biopsy‐naïve men with PSA < 10 ng/mL; therefore, it has *restrictive* intended clinical use (**C6**). Notably, the biomarker has not been validated in patients undergoing repeat biopsy or with prior negative biopsy.

### 3.4 ExoDx

ExoDx has been validated in two prospective multi‐center studies for biopsy‐naïve patients each enrolling approximately 500 patients for the intended use population and meeting our definition of a validation study.^7^, ^17^ Additionally, both studies predicted more than 50 high‐grade cancers would be detected above the recommended cut‐off point of 15.6; however, due to the limited number of validation studies (two), ExoDx did not meet criteria for wide external validation (**C1**). Another study evaluating the clinical utility of ExoDx in 458 men was not considered to be a validation study as biopsy outcome was not blinded to biomarker utilization.^18^ Furthermore, neither validation study included ≥100 non‐White patients (**C2**).

As no data from a meta‐analysis was available, discriminative accuracy for high‐grade prostate cancer was determined from the validation study enrolling the largest number of patients for the intended use population (*n* = 519); the discriminative accuracy of ExoDx was *medium* (AUC 0.73, 95% CI, 0.68–0.77) (**C8**).^19^ In the only real‐world impact study, use of ExoDx decreased biopsy utilization when below the cut‐off point and increased biopsy utilization when above the cut‐off point. Overall, use of ExoDx increased biopsy utilization over a blinded control arm (58% vs. 39%) without an undue reduction in high‐grade cancers detected.^18^ The study cautions that the observed increase in prostate biopsies should be interpreted in the context of a control arm with an unexplainably poor biopsy compliance rate when compared to earlier historic trends; as a result, the diagnostic utility of ExoDx in clinical practice was considered *unclear* (**C9**).

### 3.5 MyProstateScore

Despite reporting a separate risk stratification for prior negative biopsy,^20^ MPS has not been validated in this setting nor for PSA > 10 ng/mL and therefore meets our criteria for *restrictive* intended clinical use (**C6**).

No meta‐analyses of MPS exist. MPS was determined to have *high* discriminative accuracy for high‐grade prostate cancer (AUC 0.77 [95% CI not reported]) from the validation study enrolling the largest number of patients (*n* = 1225) for the intended use population (**C8**).^21^ While other studies have reported higher levels of discriminative accuracy, they do so by incorporating MPS into a base model that includes variables from either the PCPT^22^, ^23^ (DRE, age, African‐American race, history of prior negative biopsy and first‐degree family history of prostate cancer) or ERSPC (DRE, transrectal ultrasound and prostate volume) risk calculators. The only study evaluating the diagnostic utility of MPS in clinical practice did not include outcomes on men who were not offered biomarker testing and did not include a historic or contemporary control. Therefore, this characteristic was determined to be *not done* (**C9**).

## 4 DISCUSSION

We evaluated five commonly used diagnostic prostate cancer biomarkers—*phi*, 4KScore, SelectMDx, ExoDx and MPS—utilizing a novel framework enabling meaningful comparison of biomarker *validity* (**C1–2**), *usability* (**C3–6**) and *utility* (**C7–10**).

Previously, there has been no framework for meaningful comparison between diagnostic prostate cancer biomarkers. Moreover, very few well‐designed studies have attempted to directly compare biomarkers at all. In a comparison of SelectMDx versus 4KScore, SelectMDx had poorer discrimination of high‐grade prostate cancer than 4KScore (AUC 0.672 vs. 0.830) in a study of 114 American men (21% non‐White).^24^ This contrasts with our determination of SelectMDx possessing *very high* discriminative accuracy,^25^ which may be attributed to SelectMDx validation in a population primarily of Caucasian, European men and not in non‐White populations. In comparison of ExoDx versus 4KScore, only 8% of men received both biomarkers, and magnetic resonance imaging (MRI) determined receipt of biopsy.^19^ These are the only two studies providing comparative evidence between biomarkers; however they do so only regarding discriminative accuracy as an outcome. Taken together with existing exhaustive reviews of biomarkers, one can clearly appreciate the absence of a practical, summative and standardized assessment requisite for intelligible comparison between diagnostic prostate cancer biomarkers.

Our study has important implications for clinical practice. Our framework enables us to conclude that 4KScore demonstrates the best overall features as a diagnostic prostate cancer biomarker, a finding also supported by the few comparative studies of 4KScore with SelectMDx and ExoDx. For urine‐based tests, there are evident trade‐offs: ExoDx has better *usability* (non‐invasive, evidence in prior negative biopsy) than MPS, but with the trade‐off of worse *validity* (not validated widely or in non‐White populations) and *utility* (poorer discriminative accuracy). This framework enables providers to easily tailor a biomarker to their practice. For example, a provider with a large non‐White practice would do better with 4KScore versus SelectMDx as a blood‐based test. Alternatively, a provider interested in at‐home ExoDx collection would know this added convenience comes at the expense of discriminative accuracy when compared to MPS. With so many biomarkers to choose from, our standardized framework for providers to appropriately select a biomarker to inform decision making regarding elevated PSAs.

Future studies on diagnostic prostate cancer biomarkers should focus on several key areas. First, there needs to be an increased focus on validation in non‐White populations (e.g., SelectMDx, ExoDx) and broadened intended clinical use (e.g., SelectMDx, MPS). Second, more well‐designed studies evaluating clinical utility are needed for all biomarkers—our assessment of diagnostic utility referenced just four studies. Standardization in nomenclature and study design is similarly lacking: In the literature, ‘clinical utility’ studies varyingly describe studies where all men were biopsied,^26^, ^27^, ^28^ biomarker utility was confounded by MRI,^19^, ^29^ and lack of a control group prevented evaluation of changes in prostate cancer incidence.^16^ Well‐designed, standardized clinical utility studies would also allow for fairer comparison between biomarkers. Finally, more comparative studies should be encouraged, particularly between biomarkers with little biologic overlap (e.g., 4KScore vs. SelectMDx) or similar sampling type (e.g., ExoDx vs. MPS).

Our study should be interpreted in the context of the novelty of our study design. First, our biomarker characteristics and their evaluative criteria were deductively ascertained; they purport to be neither validated measures nor broadly inclusive. However, we assert that all of our biomarker characteristics are reasonable, transparent and granular enough to enable meaningful biomarker comparison, especially given that no similar framework currently exists and our methodology was reviewed by experts. Second, our conclusions are reflective of available evidence. For example, our evaluation that ExoDx has less discriminative accuracy than MPS was determined by comparing validation studies with different populations^17^, ^21^; unfortunately, as previously discussed, the strength of such a determination is currently limited by the dearth of comparative biomarker studies. Nonetheless, the relative performance across all biomarker characteristics sufficiently outlines prostate cancer biomarker ‘quality’ to enable the conclusion that one of two biomarkers may be ‘better’ for a given patient, which ultimately carries significant practice implications. Third, our systematic search was conducted through September 2022; a full re‐review was beyond the scope of this study, but we extended this update to the other four biomarkers as well: we incorporated subsequently published literature directly bearing on our phi assessment—including population‐screening and threshold‐optimization studies supporting a lower, ~27 cut‐off and a validated nomogram incorporating phi with multiparametric MRI—along with a 2023–2024 population‐modelling analysis and MRI‐combination cohort reinforcing 4KScore's possibly beneficial (C9) rating and a 2023 randomized‐trial follow‐up bearing on ExoDx's unclear (C9) rating (Appendix S2); we are not aware of comparable post‐2022 evidence for SelectMDx or MPS that would alter our assessments.

The recent, rapid development of novel diagnostic prostate cancer biomarkers marks a turning point in efforts to improve detection of high‐grade disease, offer more personalized care and better inform decisions of whether to biopsy. However, the sheer number and diversity of available biomarkers complicate efforts to facilitate their use in practice. For the first time, our proposed framework enables the meaningful comparison between biomarkers to facilitate informed decision making for patients and providers considering prostate biopsy. The 4KScore presently demonstrates the best overall features as a diagnostic prostate cancer biomarker. However, more clinical utility, comparative and non‐White population validation studies are needed.

## 5 CONCLUSIONS

Our framework enables meaningful comparison of diagnostic prostate cancer biomarkers using criteria most salient to patients and clinicians. We demonstrate that the 4KScore currently possesses the best overall features; despite emerging evidence, more clinical utility, comparative and non‐White population validation studies are needed.

## AUTHOR CONTRIBUTIONS

*Conceptualization study design*: Ashwin Ramaswamy, Jim C. Hu. *Data acquisition*: Ashwin Ramaswamy and Tenny R. Zhang. *Data analysis and data interpretation*: Shu Wang, Behnam Nabavizadeh, Ashwin Ramaswamy, Tenny R. Zhang, Michael Tzeng and Jim C. Hu. *Manuscript drafting*: Shu Wang, Behnam Nabavizadeh and Ashwin Ramaswamy. All authors critically revised the manuscript, approved the final version and agree to be accountable for all aspects of the work.

## CONFLICT OF INTEREST STATEMENT

The authors declare no conflict of interest.

## Supporting information


**Appendix S1:** Comparing Diagnostic Prostate Cancer Biomarkers Using a Framework of Clinically Meaningful Criteria.

**Table S1.** Systematic Literature Search Criteria.

## Data Availability

The data that support the findings of this study are available from the corresponding author upon reasonable request.
