# Supplementary material for: Comparing diagnostic prostate cancer biomarkers using a framework of clinically meaningful criteria
Source: BJUI Compass. 2026 Sep 22;7(9):e70275. doi: 10.1002/bco2.70275 (PMC13595018; doi:10.1002/bco2.70275)
Supplement: Supplementary file 1 [file BCO2-7-e70275-s001.docx]

**APPENDIX: Comparing Diagnostic Prostate Cancer Biomarkers Using a Framework of Clinically Meaningful Criteria**

**(1) BIOMARKER CHARACTERISTICS AND THEIR ACCOMPANYING CRITERIA**

Biomarker *validity* reflects the extent to which the biomarker has been studied in a large number of patients. A validation study was defined as one in which biomarker utilization was blinded to biopsy outcome, high-grade prostate cancer on biopsy (i.e., ≥ grade group [GG] 2) was the endpoint in a screening population of biopsy-naïve men (i.e., ages ≥ 50 years, PSA of 2-10 ng/mL) where fusion-guided prostate biopsy was not used. Characteristics designated most salient to biomarker *validity* include:

(C1) *Wide external validation (Yes / No)*: A biomarker with one or more validation cohort increases the likelihood of expected clinical utility in heterogeneous external cohorts and provides strong evidence that the test performs as intended in clinical practice. A biomarker was considered to possess “wide external validation” if the validation cohort in at least one study meeting the criteria above included ≥ 2,000 patients, or if ≥ 50 high-grade prostate cancers were detected in at least three studies.
(C2) *Validation in non-White populations (Broad / Black / Asian / Latino / None)*: The majority of validation studies have been performed in White populations which limits generalizability of their conclusions to non-White populations. A biomarker was considered to be validated in non-White populations if the validation cohort in at least one study included ≥ 100 patients self-identifying as Black, Asian, and/or Latino, respectively; if a biomarker was validated in more than one non-White population, it was considered to have *broad* validation in non-White populations.

Biomarker *usability* reflects the extent to which the biomarker individualizes predictive modelling of disease risk and optimizes patient concerns in clinical practice. Characteristics designated most salient to biomarker *usability* include:

(C3) *Incorporates an individual’s clinical data into score (Yes / No)*: A biomarker incorporating individual clinical data (e.g., Black race, positive digital rectal exam [DRE], prior negative biopsy) into its score enables providers to personalize decisions regarding biopsy by including additional prognostic factors that would otherwise be impossible to objectively reconcile were the biomarker serum or urinary values reported in isolation.
(C4) *Reported as an individual’s probability of disease (Yes / No):* A biomarker that is calibrated to report results as an individual’s probability of disease (e.g., “your personal risk of prostate cancer is 8%”) enables easy interpretation of results by provider and patient; in contrast, biomarkers which utilize a cut-off value alone cannot provide clinical meaning to differences in the magnitude of positive or negative results (e.g., a minimally vs. elevated positive result).
(C5) *Non-invasive sampling type (Yes / No)*: A biomarker that is collected non-invasively improves the odds of biomarker utilization in clinical practice. A biomarker was considered “non-invasive” if it was measured via serum or urine samples without requiring DRE; by contrast, “invasive” biomarkers included terminology of “prostate massage” in physician instructions^1^ or required prostate volume calculation via transrectal ultrasound.
(C6) *Intended clinical use (Broad / Some / Restrictive)*: A biomarker has improved usability if it is validated for use in alternative clinical scenarios other than typical screening. Intended clinical use was determined on the basis of biomarker validation studies, not regulatory approval. We focused specifically on two common scenarios: (1) PSA > 10 ng/mL and (2) men with prior negative biopsy. Positive DRE was not included as a component of this criterion. While most biomarker validation studies included men with a positive or suspicious DRE,^2^ few report the biomarker’s discriminative accuracy in this specific subset in which weaker diagnostic performance is expected.^3^ Therefore, a biomarker was considered to possess *restrictive* intended clinical use if it was validated only in biopsy-naïve men with a PSA of 2-10 ng/mL; *narrow* intended clinical use if the biomarker was validated for use in patients with either PSA > 10 ng/mL *or* prior negative biopsy; and *broad* intended clinical use if evidence validates use of the biomarker in patients with PSA > 10 ng/mL *and* prior negative biopsy.

Biomarker *utility* assesses the extent to which a biomarker is clinically useful and performs as intended by improving discrimination of high-grade prostate cancer with the ultimate goal of preventing metastasis and mortality. Characteristics designated most salient to biomarker *utility* include:

(C7) *High-grade measured as clinical endpoint* *(Yes / No)*: Improving the detection of high-grade, clinically significant cancer while decreasing the detection of low-grade, clinically insignificant disease is the goal of prostate cancer screening. A biomarker that is validated to discriminate for high-grade prostate cancer specifically, in contrast to all prostate cancer, meets this goal.
(C8) *Discriminative accuracy for high-grade prostate cancer (Very high / High / Medium / Low):* Better discriminative ability for high-grade prostate cancer increases provider confidence in recommending for or against prostate biopsy. A biomarker’s performance in discriminating between a negative biopsy result and high-grade prostate cancer was evaluated by the area under the curve (AUC); validation studies reporting AUCs which included standard of care variables (e.g., the Prostate Cancer Prevention Trial [PCPT] risk calculator) were excluded if the biomarker did not depend on these variables. Discriminative accuracy was rated as *very high* if reported ≥ 0.8, *high* if reported between 0.75 – 0.799, *medium* if reported between 0.7 – 0.749, and *low* if < 0.7.

     If data from a meta-analysis was available, discriminative accuracy for high-grade prostate cancer was derived from a hierarchical summary receiver operator characteristic only if the meta-analysis included validation studies meeting our above criteria, and where the AUC included only the variables for which the biomarker was validated in clinical practice. Additionally, the meta-analysis needed to adhere to PRIMSA and/or evaluated studies for risk of bias using the Quality Assessment of Diagnostic Accuracy Studies (QUADAS) tool.^4^ If multiple meta-analyses met the above criteria for a given biomarker, the meta-analysis was selected based on how clearly and detailed the AUC was calculated with respect to different patient factors (e.g., studies did or did not perform a DRE), whether sensitivity analyses were included to account for heterogeneity or outliers, and whether the most recent validation studies were included. If data from a meta-analysis was not available or did not meet the above criteria, discriminative accuracy for high-grade prostate cancer was derived from the AUC reported in a validation study that met the applicable above criteria *and* included the largest number of patients. Furthermore, studies in which a biomarker was used conjointly with another test (e.g., MRI) were included if they provided a discrete estimate of discrimination for the biomarker itself. We excluded articles in which the primary results were reported in terms of sensitivity or specificity without a specified cut-off value or where the AUC of the biomarker was not specifically reported.
(C9) *Diagnostic utility in clinical practice (Likely beneficial / Possibly beneficial / Likely harmful / Possibly harmful / Unclear / Not done)*: While a biomarker may demonstrate improved discriminative accuracy in a validation study, it may have unintended and possibly deleterious effects in real-world clinical practice. Ideally, a biomarker with increased utility in clinical practice should (1) decrease prostate biopsy rates and (2) decrease detection of low-grade prostate cancer without compromising (3) detection of high-grade prostate cancer, when compared to current standard of care practice patterns.

     As such, we reviewed clinical utility studies in which men being evaluated for prostate cancer received biomarker testing and biopsy per provider discretion. The (1) *change* in prostate biopsy rates was determined; additionally, the (2) *change* in low-grade or (3) high-grade prostate cancer incidence was determined if a historic or prospective control group was used.

     A biomarker was considered to have *likely beneficial* diagnostic utility if it achieved all three criteria; conversely, a biomarker was considered to have *likely harmful* diagnostic utility if it was associated with an undue observed reduction in high-grade cancer detection.

     In the absence of a historic or prospective control, the *change* in prostate cancer incidence was unable to be determined. However, given that all studies included men whom presumably would have received a biopsy without the biomarker, a biomarker associated with a reduction vs. increase in prostate biopsy rates was considered to have *possibly beneficial* vs. *harmful* diagnostic utility, respectively.

     If a clinical utility study did not meet the above criteria or was performed with clear confounders, it was considered *unclear*. If no clinical utility studies met our inclusion criteria or if it did not report on all of three outcomes, it was considered *not done*.
(C10) *Oncologic endpoints in clinical practice (Yes / No)*: Early detection of high-grade disease is a priority as it is a surrogate for the consequential clinical endpoints of prostate cancer metastasis and mortality. A biomarker was considered to be validated for oncologic endpoints in clinical practice if it was demonstrated to improve discrimination of metastasis or disease-specific survival in a study.

**(2) ASSESSMENT OF *phi*, 4KScore, ExoDx, SelectMDx, & MPS**

*Prostate Health Index*

The *phi* is a combination of three PSA isoforms: total PSA, free PSA, and [-2]proPSA. The *phi* met our criterion for wide validation (**C1**) in an international multicenter study evaluating 1,688 Asian men and 800 European men.^5^ *phi* was considered to possess broad validation in non-White populations (**C2**) including Black,^6^ Hispanic,^7^ and Arabic^8,9^ populations; notably, *phi* has been validated in a large number of Asian populations.^5,10–13^

The *phi* does not incorporate clinical data into its score (**C3**). An optimal *phi* cutoff is not clearly defined nor standardized and remains controversial with the manufacturer presently reporting results in four distinct risk thresholds (0-26.9, 27.0-35.9, 36.0-54.9, and 55.0+) that represent an increasing probability of prostate cancer (**C4**); *phi* cutoffs indicating a positive test are usually between 24 and 40.^14^ The *phi* is a non-invasive blood test (**C5**) and has been *broadly* studied in different clinical use cases (**C6**) including repeat biopsy^5^ (including prior negative biopsy^15^) and PSA > 10 ng/mL^16^

The *phi* as designed predicts the patient’s probability of having any prostate cancer; it does not report high-grade prostate cancer as a clinical endpoint (**C7**) to providers ordering the test. The *phi* was determined to have *very high* discriminative accuracy for high-grade prostate cancer determined from a meta-analysis of seven clinical validation studies (compared to 23 studies evaluating *all* prostate cancer) comprising a total of 3,227 patients reporting an AUC of 0.82 (95% CI, 0.70-0.93),^17^ (**C8**) improving on a previous meta-analysis^18^ by an additional four studies. Of note, while several other meta-analyses were performed that met our criteria, none uniquely reported discrimination of high-grade prostate cancer. Importantly, it appears that the *very high* discriminative accuracy of *phi* does not necessary carry over to non-White populations.^6,19^

For *phi*, characteristic **(C9)** “Diagnostic utility in clinical practice” was determined as *Unclear*. Three studies have evaluated the diagnostic utility of *phi* in real clinical practice. White et al. reported on the clinical utility of *phi* in 506 men across four large urology practices compared to historical controls. In this study, use of *phi* resulted in a significant reduction in biopsies performed (36% *phi* vs. 60% historic) as well as an overall reduction in the detection of low-grade prostate cancer (10% vs. 18%).^20^ Notably, all men received the *phi* test; use was not based on provider discretion. However, further analysis by Ehdaie et al. in a correspondence letter noted one high-grade cancer was missed for about every three biopsies not performed.^21^ Moreover, 39 patients initially planned for biopsy were switched to surveillance despite receiving an “elevated risk” *phi* score of 36-55+; this may indicate providers face challenges in understanding and implementing *phi*’s risk thresholds in clinical practice. In their response, White et al. presented additional data demonstrating a 7% decrease in high-grade prostate cancer detection from 18.3% to 11.3% in the historic and prospective cohorts, respectively, in contrast to an 8.5% decrease in GG1 detection. By contrast, a comparable single-institution study that relied on provider discretion rather than a fixed *phi* threshold found a 9% reduction in prostate biopsies driven by decreased detection of negative and low-grade (GG1) disease, without a corresponding decrease in high-grade (GG2+) cancer detection.^22^ This discrepancy may reflect that White et al.’s ≥36 cutoff for “elevated risk” exceeds the ~27 threshold supported by other validation and population-screening studies as an appropriate balance of sensitivity and specificity for high-grade disease, rather than reflecting an inherent limitation of *phi*.^23,24^ Incorporation of *phi* into nomograms alongside multiparametric MRI has since been shown to further improve detection of clinically significant disease while reducing biopsies.^25^ Given these conflicting findings—and given that the same absence of a historic control did not preclude a *possibly beneficial* (C9) rating for 4KScore—the diagnostic utility of *phi* in clinical practice was determined to be Unclear rather than likely harmful. Lastly, two studies demonstrate that high *phi* risk score is an independent predictor of lymph node^26^ and bone^27^ metastasis (**C10**).

*4Kscore*

The 4K Score is a model incorporating four kallikrein biomarkers (total PSA, free PSA, intact PSA, and human kallikrein-related peptidase 2) and patient age, DRE, and history of prior prostate biopsy. The 4K Score possesses wide external validation (**C1**) as it has been validated in a prospective study measuring those four kallikrein biomarkers in cryopreserved blood plasma samples of 4,765 patients originally enrolled in the large randomized prospective ProtecT study.^28^ While this validation study did not include DRE or history of prior prostate biopsy as does the commercial 4K Score, additional studies with models predicting ≥ 50 high-grade cancers detected address these discrepancies.^29–32^ The biomarker was also validated in a prospective study at eight Veterans Affairs medical centers in which the majority of the intended use population identified as African American (205 men). While one retrospective study of a large population of Latino and Asian men in the Multiethnic Cohort demonstrated a high discriminative accuracy (AUC 0.77 [0.75 – 0.81]) for the detection of aggressive prostate cancer, this study did not meet our criteria for a validation study as not all participants were biopsied, high-grade prostate cancer was not used as a primary outcome, and the authors were unable to identify any men with Gleason 7 disease due to unforeseen data limitations.^33,34^ As a result, the 4K Score was considered to be validated in Black patients only (**C2**).

The 4K Score incorporates quantification of four serum kallikreins (**C5**) with age, DRE, and history of prior prostate biopsy (**C3**) and is reported as a range from 0-100%, reflecting the probability that a patient will have high-grade prostate cancer on biopsy (**C4**). The 4K Score was considered to have *broad* intended clinical use (**C6**) since it has been validated in men with prior negative prostate biopsy^35^ and men with a recent DRE,^36^ positive DRE, or a PSA of 10-25 ng/mL.^2^

The 4K Score report presents a personalized risk score that reflects the probability a patient will have high-grade prostate cancer on biopsy with a commonly used threshold of 7.5% to guide decision making (**C7**).^37^ The 4K Score was determined to have *very high* discriminative accuracy for high-grade prostate cancer determined from a meta-analysis of 12 clinical validation studies comprising a total of 11,134 patients reporting an AUC of 0.81 (95% CI, 0.80-0.83) (**C8**); this meta-analysis accounted for models with and without DRE and included a sensitivity analysis excluding outliers and cohorts not part of the European Research Study on Prostate Cancer (ERSPC).^38^ A second meta-analysis of 28 studies comprising a total of 16,762 patients similarly reported an AUC of 0.81 (95% CI, 0.77-0.85); this meta-analysis was not selected as it did not specify strict inclusion of validation studies, detail how DRE was accounted for, or utilize sensitivity analyses.^17^

Although a clinical utility study was performed evaluating the 4KScore demonstrating a 65% decrease in biopsies, the absence of a historic or prospective control group prevents interpretation of the resultant change in incidence of low-grade and high-grade prostate cancer.^39^ However, as authors reported the distribution of 4KScore results in non-biopsied patients and that the 4KScore can be interpreted as the probability of high-grade prostate cancer, some inference on cancer outcomes is possible. Therefore, the diagnostic utility of this biomarker in clinical practice was considered *possibly beneficial* (**C9**). This determination is reinforced by subsequent real-world evidence: a US population-modeling analysis estimated that 4KScore implementation leads to approximately 45,200 fewer biopsies and 9,400 fewer low-grade overdiagnoses annually, at the cost of delayed diagnosis of high-grade disease in an estimated 3,450 men.^40^ A separate retrospective cohort further found that 4KScore remained independently associated with the decision to pursue biopsy and improved discrimination for high-grade disease (GG2+) when used alongside MRI.^41^ Lastly, a retrospective case-control study comparing baseline PSA with the four-kallikrein panel in 11,506 men who were followed without PSA screening demonstrated that the four-kallikrein panel increased the discrimination for cancer-specific mortality by 0.07 (95% CI 0.04, 0.10) from 0.73 to 0.80 (**C10**).^42^

*SelectMDx*

The SelectMDx test is a urine-based biomarker incorporating post-DRE mRNA levels of the HOXC6 and DLX1 genes combined with clinical characteristics such as patient age, DRE, and PSA density (**C3**). The SelectMDx test was validated in three studies meeting our above criteria in 386,^43^ 715,^44^ and 599^45^ European men, respectively, all of which detected more than 50 high-grade cancers above the recommended cut-off point of -2.8; therefore, SelectMDx met our criteria for wide external validation (**C1**). Two other validation studies did not meet criteria as they included men who all received MRI and subsequent fusion-guided biopsy, rather than systematic biopsy.^46,47^ An additional validation study did not meet criteria as SelectMDx score influenced decision to biopsy.^48^ Currently there are no validation studies in non-White populations (**C2**).

SelectMDx is reported as a continuous risk score of -6 to 6, where a more positive number is associated with an increased likelihood that biopsy would identify high-grade prostate cancer (**C7**) with a binary cutoff for a positive test set at -2.8 (**C4**).^44^ Furthermore, as SelectMDx requires post-DRE prostate massage as well as PSA density (via prostate volume calculated by transrectal ultrasound), this biomarker was determined to have an invasive sampling type (**C5**). SelectMDx has only been validated in biopsy-naïve men with PSA < 10 ng/mL, therefore has *restrictive* intended clinical use (**C6**); notably, the biomarker is not validated in patients undergoing repeat biopsy or with prior negative biopsy.

SelectMDx was determined to have *very high* discriminative accuracy for high-grade prostate cancer (AUC 0.82 [95% CI, 0.79-0.86]) from the validation study enrolling the largest number of patients for the intended use population, in this case 715 European men (**C8**). A recent meta-analysis on SelectMDx used flawed search criteria by not including the terms “HOXC6” or “DLX1” and therefore did not identify either of the above validation studies in its analysis.^49^ There are no impact studies in real-world clinical practice addressing either diagnostic (**C9**) or oncologic (**C10**) endpoints.

*ExoDx*

ExoDx has been validated in two prospective multi-center studies for biopsy-naïve patients each enrolling approximately 500 patients for the intended use population and meeting our above definition of a validation study.^3,50^ Additionally, both studies predicted more than 50 high-grade cancers would be detected above the recommended cut-off point of 15.6; however, due to the limited number of validation studies (two), ExoDx did not meet our criteria for wide external validation (**C1**). Another study evaluating the clinical utility of ExoDx in 458 men was not considered to be a validation study as biopsy outcome was not blinded to biomarker utilization.^27^ Furthermore, neither validation study included ≥ 100 non-White patients (**C2**).

This biomarker is calculated based on the relative gene expression signature from PCA3 and ERG RNA detected in urine (**C5**) without inclusion of other clinical parameters (**C3**). The test is reported as a risk score from 0-100 with a recommended cut-off of 15.6 without reporting a predicted probability of disease (**C4**). The biomarker was considered to have *broad* intended clinical use (**C6**) given it has been validated for men with PSA > 10 ng/mL^3^ and prior negative biopsy.^51^

Reports from an ExoDx assay specifically state that an individual is at higher risk of high-grade prostate cancer after prostate biopsy if the result is above the cut-off value of 15.6 (**C7**). As no data from a meta-analysis was available, discriminative accuracy for high-grade prostate cancer was determined from the validation study enrolling the largest number of patients for the intended use population, in this case 519 patients; the discriminative accuracy of ExoDx was *medium* (AUC 0.73, 95% CI, 0.68-0.77) (**C8**).^26^ In the only real-world impact study, use of ExoDx decreased biopsy utilization when below the cut-off point and increased biopsy utilization when above the cut-off point; overall, use of ExoDx increased biopsy utilization over a blinded control arm (58% vs. 39%) without an undue reduction in high-grade cancers detected.^52^ The study cautions that the observed increase in prostate biopsies should be interpreted in the context of a control arm with an unexplainably poor biopsy compliance rate when compared to earlier historic trends; as a result, the diagnostic utility of ExoDx in clinical practice was considered *unclear* (**C9**). A 2023 interim analysis of the underlying randomized Decision Impact Trial (2.5-year follow-up) reported that men who received low-risk EPI results deferred biopsy significantly longer than those who did not, and that clinicians with access to EPI results identified 21.8% more high-grade cancer than those relying on standard of care alone, while low-risk EPI patients remained at very low pathologic risk over follow-up.^53^ However, because the trial’s intervention arm underwent more, not fewer, biopsies overall than the control arm, this study does not resolve the C9 designation and the diagnostic utility of ExoDx in clinical practice remains *unclear*. Lastly, there is no reported evidence of oncologic endpoints in clinical practice (**C10**).

*MyProstateScore*

The MyProstateScore (MPS) test (also termed Mi-Prostate Score or MiPS) combines PSA with PCA3 (a noncoding RNA) and the TMPRSS2:ERG fusion oncogene, both of which are specific for prostate cancer. MPS has been widely validated (**C1**) in three large studies where more than 50 high-grade cancers were detected in each.^54–56^ Additionally, MPS has been validated in Black men (**C2**) in a large prospective cohort where 60% were African American;^57^ analysis in other ethnic cohorts has not been done.

MPS does not depend on clinical variables outside PSA, PCA3, and TMPRSS2:ERG (**C3**). MPS ranges from 0 to 100, with higher scores reflecting increased risk of high-grade prostate cancer on biopsy (**C4, C7**); utilizing a cutoff at 10.0 for decision making, MPS stratifies patients into low- (≤ 10.0), intermediate- (10.1-25.0), or high-risk (> 25.0) groups for biopsy-naïve patients. PCA3 and TMPRSS2:ERG are both urinary biomarkers collected after prostate massage (**C5**). Despite reporting a separate risk stratification for patients with a prior negative biopsy,^58^ MPS has not been validated in this setting nor for PSA > 10 ng/mL and therefore meets our criteria for *restrictive* intended clinical use (**C6**).

No meta-analyses of MPS exist. MPS was determined to have *high* discriminative accuracy for high-grade prostate cancer (AUC 0.77 [95% CI not reported]) from the validation study enrolling the largest number of patients for the intended use population, in this case 1,225 European men (**C8**).^54^ While other studies have reported higher levels of discriminative accuracy, they do so by incorporating MPS into a base model which includes variables from either the PCPT^56,59^ (DRE, age, African-American race, history of prior negative biopsy, and first-degree family history of prostate cancer) or ERSPC (DRE, transrectal ultrasound, and prostate volume) risk calculators. The only study evaluating the diagnostic utility of MPS in clinical practice did not include outcomes on men who were not offered biomarker testing and did not include a historic or contemporary control; therefore, this characteristic was determined to be *not done* (**C9**). There are no impact studies in clinical practice addressing oncologic (**C10**) endpoints.
**REFERENCES**

1.	SelectMDx Testing – LifeLabs. Accessed December 12, 2022. https://www.lifelabs.com/test/selectmdx/

2.	Vickers A, Vertosick EA, Sjoberg DD, et al. Properties of the 4-Kallikrein Panel Outside the Diagnostic Gray Zone: Meta-Analysis of Patients with Positive Digital Rectal Examination or Prostate Specific Antigen 10 ng/ml and Above. *J Urol*. 2017;197(3 Pt 1):607-613. doi:10.1016/j.juro.2016.09.086

3.	McKiernan J, Donovan MJ, O’Neill V, et al. A Novel Urine Exosome Gene Expression Assay to Predict High-grade Prostate Cancer at Initial Biopsy. *JAMA Oncol*. 2016;2(7):882-889. doi:10.1001/jamaoncol.2016.0097

4.	Whiting PF, Rutjes AWS, Westwood ME, et al. QUADAS-2: a revised tool for the quality assessment of diagnostic accuracy studies. *Ann Intern Med*. 2011;155(8):529-536. doi:10.7326/0003-4819-155-8-201110180-00009

5.	Chiu PK, Ng CF, Semjonow A, et al. A Multicentre Evaluation of the Role of the Prostate Health Index (PHI) in Regions with Differing Prevalence of Prostate Cancer: Adjustment of PHI Reference Ranges is Needed for European and Asian Settings. *Eur Urol*. 2019;75(4):558-561. doi:10.1016/j.eururo.2018.10.047

6.	Babajide R, Carbunaru S, Nettey OS, et al. Performance of Prostate Health Index in Biopsy Naïve Black Men. *J Urol*. 2021;205(3):718-724. doi:10.1097/JU.0000000000001453

7.	Liang Y, Ankerst DP, Ketchum NS, et al. Prospective evaluation of operating characteristics of prostate cancer detection biomarkers. *J Urol*. 2011;185(1):104-110. doi:10.1016/j.juro.2010.08.088

8.	Al Saidi SS, Al Riyami NB, Al Marhoon MS, et al. Validity of Prostate Health Index and Percentage of [-2] Pro-Prostate-Specific Antigen as Novel Biomarkers in the Diagnosis of Prostate Cancer: Omani Tertiary Hospitals Experience. *Oman Med J*. 2017;32(4):275-283. doi:10.5001/omj.2017.55

9.	Nassir AM. A piece in prostate cancer puzzle: Future perspective of novel molecular signatures. *Saudi J Biol Sci*. 2020;27(4):1148-1154. doi:10.1016/j.sjbs.2020.02.003

10.	Cheng YT, Chiang CH, Pu YS, et al. The application of p2PSA% and prostate health index in prostate cancer detection: A prospective cohort in a Tertiary Medical Center. *J Formos Med Assoc*. 2019;118(1 Pt 2):260-267. doi:10.1016/j.jfma.2018.05.001

11.	Othman H, Abu Yamin AH, Md Isa N, Bahadzor B, Syed Zakaria SZ. Diagnostic performance of prostate health index (PHI) in predicting prostate cancer on prostate biopsy. *Malays J Pathol*. 2020;42(2):209-214.

12.	Abrate A, Lughezzani G, Gadda GM, et al. Clinical use of [-2]proPSA (p2PSA) and its derivatives (%p2PSA and Prostate Health Index) for the detection of prostate cancer: a review of the literature. *Korean J Urol*. 2014;55(7):436-445. doi:10.4111/kju.2014.55.7.436

13.	Hirama H, Sugimoto M, Ito K, Shiraishi T, Kakehi Y. The impact of baseline [-2]proPSA-related indices on the prediction of pathological reclassification at 1 year during active surveillance for low-risk prostate cancer: the Japanese multicenter study cohort. *J Cancer Res Clin Oncol*. 2014;140(2):257-263. doi:10.1007/s00432-013-1566-2

14.	Stojadinovic M, Vukovic I, Ivanovic M, et al. Optimal threshold of the prostate health index in predicting aggressive prostate cancer using predefined cost-benefit ratios and prevalence. *Int Urol Nephrol*. 2020;52(5):893-901. doi:10.1007/s11255-019-02367-z

15.	Rosenkrantz AB, Verma S, Choyke P, et al. Prostate Magnetic Resonance Imaging and Magnetic Resonance Imaging Targeted Biopsy in Patients with a Prior Negative Biopsy: A Consensus Statement by AUA and SAR. *J Urol*. 2016;196(6):1613-1618. doi:10.1016/j.juro.2016.06.079

16.	Nordström T, Vickers A, Assel M, Lilja H, Grönberg H, Eklund M. Comparison Between the Four-kallikrein Panel and Prostate Health Index for Predicting Prostate Cancer. *European Urology*. 2015;68(1):139-146. doi:10.1016/j.eururo.2014.08.010

17.	Russo GI, Regis F, Castelli T, et al. A Systematic Review and Meta-analysis of the Diagnostic Accuracy of Prostate Health Index and 4-Kallikrein Panel Score in Predicting Overall and High-grade Prostate Cancer. *Clin Genitourin Cancer*. 2017;15(4):429-439.e1. doi:10.1016/j.clgc.2016.12.022

18.	Wang W, Wang M, Wang L, Adams TS, Tian Y, Xu J. Diagnostic ability of %p2PSA and prostate health index for aggressive prostate cancer: a meta-analysis. *Sci Rep*. 2014;4:5012. doi:10.1038/srep05012

19.	Morris KE, Kotamarti S, Polascik TJ, Moul JW. Re-thinking How We Use Prostate Health Index for African American Men. *Urology*. Published online February 22, 2023:S0090-4295(23)00176-0. doi:10.1016/j.urology.2022.12.057

20.	White J, Shenoy BV, Tutrone RF, et al. Clinical utility of the Prostate Health Index (phi) for biopsy decision management in a large group urology practice setting. *Prostate Cancer Prostatic Dis*. 2018;21(1):78-84. doi:10.1038/s41391-017-0008-7

21.	Ehdaie B, Carlsson S. Reply to ‘Clinical utility of the Prostate Health Index (phi) for biopsy decision management in a large group urology practice setting.’ *Prostate Cancer Prostatic Dis*. 2018;21(3):446-447. doi:10.1038/s41391-018-0052-y

22.	Tosoian JJ, Druskin SC, Andreas D, et al. Use of the Prostate Health Index for detection of prostate cancer: results from a large academic practice. *Prostate Cancer Prostatic Dis*. 2017;20(2):228-233. doi:10.1038/pcan.2016.72

23.	Ito K, Yokomizo A, Tokunaga S, et al. Diagnostic Impacts of Clinical Laboratory Based p2PSA Indexes on any Grade, Gleason Grade Group 2 or Greater, or 3 or Greater Prostate Cancer and Prostate Specific Antigen below 10 ng/ml. *J Urol*. 2020;203(1):83-91. doi:10.1097/JU.0000000000000495

24.	Yaegashi S, Miwa S, et al. Clinical Validation of the Prostate Health Index for Detecting Clinically Significant Prostate Cancer in a Population-Based Screening Program. *Int J Urol*. 2026;33(3):e70409. doi:10.1111/iju.70409

25.	Siddiqui MR, Li EV, Kumar SKSR, et al. Optimizing detection of clinically significant prostate cancer through nomograms incorporating mri, clinical features, and advanced serum biomarkers in biopsy naïve men. *Prostate Cancer Prostatic Dis*. 2023;26(3):588-595. doi:10.1038/s41391-023-00660-8

26.	Nassir AM, Kamel HFM. Explication of the roles of prostate health index (PHI) and urokinase plasminogen activator (uPA) as diagnostic and predictor tools for prostate cancer in equivocal PSA range of 4-10 ng/mL. *Saudi J Biol Sci*. 2020;27(8):1975-1984. doi:10.1016/j.sjbs.2020.04.004

27.	Sun H, Zhu Y, Guo H, Jiang S, Chen S. Evaluation of prostate health index in predicting bone metastasis of prostate cancer before bone scanning. *Int Urol Nephrol*. Published online 2022. doi:10.1007/s11255-022-03340-z

28.	Bryant RJ, Sjoberg DD, Vickers AJ, et al. Predicting high-grade cancer at ten-core prostate biopsy using four kallikrein markers measured in blood in the ProtecT study. *J Natl Cancer Inst*. 2015;107(7). doi:10.1093/jnci/djv095

29.	Lin DW, Newcomb LF, Brown MD, et al. Evaluating the Four Kallikrein Panel of the 4Kscore for Prediction of High-grade Prostate Cancer in Men in the Canary Prostate Active Surveillance Study. *Eur Urol*. 2017;72(3):448-454. doi:10.1016/j.eururo.2016.11.017

30.	Parekh DJ, Punnen S, Sjoberg DD, et al. A multi-institutional prospective trial in the USA confirms that the 4Kscore accurately identifies men with high-grade prostate cancer. *Eur Urol*. 2015;68(3):464-470. doi:10.1016/j.eururo.2014.10.021

31.	Braun K, Sjoberg DD, Vickers AJ, Lilja H, Bjartell AS. A Four-kallikrein Panel Predicts High-grade Cancer on Biopsy: Independent Validation in a Community Cohort. *Eur Urol*. 2016;69(3):505-511. doi:10.1016/j.eururo.2015.04.028

32.	Vickers AJ, Cronin AM, Roobol MJ, et al. A four-kallikrein panel predicts prostate cancer in men with recent screening: data from the European Randomized Study of Screening for Prostate Cancer, Rotterdam. *Clin Cancer Res*. 2010;16(12):3232-3239. doi:10.1158/1078-0432.CCR-10-0122

33.	Darst BF, Chou A, Wan P, et al. The Four-Kallikrein Panel Is Effective in Identifying Aggressive Prostate Cancer in a Multiethnic Population. *Cancer Epidemiol Biomarkers Prev*. 2020;29(7):1381-1388. doi:10.1158/1055-9965.EPI-19-1560

34.	Punnen S, Freedland SJ, Polascik TJ, et al. A Multi-Institutional Prospective Trial Confirms Noninvasive Blood Test Maintains Predictive Value in African American Men. *J Urol*. 2018;199(6):1459-1463. doi:10.1016/j.juro.2017.11.113

35.	Gupta A, Roobol MJ, Savage CJ, et al. A four-kallikrein panel for the prediction of repeat prostate biopsy: data from the European Randomized Study of Prostate Cancer screening in Rotterdam, Netherlands. *Br J Cancer*. 2010;103(5):708-714. doi:10.1038/sj.bjc.6605815

36.	Maccini MA, Westfall NJ, Van Bokhoven A, et al. The effect of digital rectal exam on the 4Kscore for aggressive prostate cancer. *Prostate*. 2018;78(7):506-511. doi:10.1002/pros.23495

37.	Mi C, Bai L, Yang Y, Duan J, Gao L. 4Kscore diagnostic value in patients with high-grade prostate cancer using cutoff values of 7.5% to 10%: A meta-analysis. *Urol Oncol*. 2021;39(6):366.e1-366.e10. doi:10.1016/j.urolonc.2020.11.001

38.	Zappala SM, Scardino PT, Okrongly D, Linder V, Dong Y. Clinical performance of the 4Kscore Test to predict high-grade prostate cancer at biopsy: A meta-analysis of us and European clinical validation study results. *Rev Urol*. 2017;19(3):149-155. doi:10.3909/riu0776

39.	Konety B, Zappala SM, Parekh DJ, et al. The 4Kscore® Test Reduces Prostate Biopsy Rates in Community and Academic Urology Practices. *Rev Urol*. 2015;17(4):231-240.

40.	Scuderi S, Tin A, Gandaglia G, Stabile A, Montorsi F, Briganti A, Vickers AJ. Implementation of 4Kscore as a Secondary Test Before Prostate Biopsy: Impact on US Population Trends for Prostate Cancer. *Eur Urol Open Sci*. 2023;52:1-3. doi:10.1016/j.euros.2023.03.011

41.	Wang J, et al. The Clinical Impact of the 4Kscore Test on Prostate Biopsy Decision Making in the Setting of MRI. *JU Open Plus*. 2024;2(4):e00028. doi:10.1097/JU9.0000000000000134

42.	Sjoberg DD, Vickers AJ, Assel M, et al. Twenty-year Risk of Prostate Cancer Death by Midlife Prostate-specific Antigen and a Panel of Four Kallikrein Markers in a Large Population-based Cohort of Healthy Men. *Eur Urol*. 2018;73(6):941-948. doi:10.1016/j.eururo.2018.02.016

43.	Van Neste L, Hendriks RJ, Dijkstra S, et al. Detection of High-grade Prostate Cancer Using a Urinary Molecular Biomarker-Based Risk Score. *Eur Urol*. 2016;70(5):740-748. doi:10.1016/j.eururo.2016.04.012

44.	Haese A, Trooskens G, Steyaert S, et al. Multicenter Optimization and Validation of a 2-Gene mRNA Urine Test for Detection of Clinically Significant Prostate Cancer before Initial Prostate Biopsy. *J Urol*. 2019;202(2):256-263. doi:10.1097/JU.0000000000000293

45.	Hendriks RJ, van Oort IM, Schalken JA. Blood-based and urinary prostate cancer biomarkers: a review and comparison of novel biomarkers for detection and treatment decisions. *Prostate Cancer Prostatic Dis*. 2017;20(1):12-19. doi:10.1038/pcan.2016.59

46.	Roumiguié M, Ploussard G, Nogueira L, et al. Independent Evaluation of the Respective Predictive Values for High-Grade Prostate Cancer of Clinical Information and RNA Biomarkers after Upfront MRI and Image-Guided Biopsies. *Cancers (Basel)*. 2020;12(2). doi:10.3390/cancers12020285

47.	Maggi M, Del Giudice F, Falagario UG, et al. SelectMDx and Multiparametric Magnetic Resonance Imaging of the Prostate for Men Undergoing Primary Prostate Biopsy: A Prospective Assessment in a Multi-Institutional Study. *Cancers (Basel)*. 2021;13(9). doi:10.3390/cancers13092047

48.	Wysock JS, Becher E, Persily J, Loeb S, Lepor H. Concordance and Performance of 4Kscore and SelectMDx for Informing Decision to Perform Prostate Biopsy and Detection of Prostate Cancer. *Urology*. 2020;141:119-124. doi:10.1016/j.urology.2020.02.032

49.	Sari Motlagh R, Yanagisawa T, Kawada T, et al. Accuracy of SelectMDx compared to mpMRI in the diagnosis of prostate cancer: a systematic review and diagnostic meta-analysis. *Prostate Cancer Prostatic Dis*. 2022;25(2):187-198. doi:10.1038/s41391-022-00538-1

50.	McKiernan J, Donovan MJ, Margolis E, et al. A Prospective Adaptive Utility Trial to Validate Performance of a Novel Urine Exosome Gene Expression Assay to Predict High-grade Prostate Cancer in Patients with Prostate-specific Antigen 2-10ng/ml at Initial Biopsy. *Eur Urol*. 2018;74(6):731-738. doi:10.1016/j.eururo.2018.08.019

51.	McKiernan J, Noerholm M, Tadigotla V, et al. A urine-based Exosomal gene expression test stratifies risk of high-grade prostate Cancer in men with prior negative prostate biopsy undergoing repeat biopsy. *BMC Urol*. 2020;20(1):138. doi:10.1186/s12894-020-00712-4

52.	Tutrone R, Donovan MJ, Torkler P, et al. Clinical utility of the exosome based ExoDx Prostate(IntelliScore) EPI test in men presenting for initial Biopsy with a PSA 2-10 ng/mL. *Prostate Cancer Prostatic Dis*. 2020;23(4):607-614. doi:10.1038/s41391-020-0237-z

53.	Tutrone R, Lowentritt B, Neuman B, et al. ExoDx prostate test as a predictor of outcomes of high-grade prostate cancer - an interim analysis. *Prostate Cancer Prostatic Dis*. 2023;26(3):596-601. doi:10.1038/s41391-023-00675-1

54.	Tomlins SA, Day JR, Lonigro RJ, et al. Urine TMPRSS2:ERG Plus PCA3 for Individualized Prostate Cancer Risk Assessment. *Eur Urol*. 2016;70(1):45-53. doi:10.1016/j.eururo.2015.04.039

55.	Tosoian JJ, Trock BJ, Morgan TM, et al. Use of the MyProstateScore Test to Rule Out Clinically Significant Cancer: Validation of a Straightforward Clinical Testing Approach. *J Urol*. 2021;205(3):732-739. doi:10.1097/JU.0000000000001430

56.	Sanda MG, Feng Z, Howard DH, et al. Association Between Combined TMPRSS2:ERG and PCA3 RNA Urinary Testing and Detection of Aggressive Prostate Cancer. *JAMA Oncol*. 2017;3(8):1085-1093. doi:10.1001/jamaoncol.2017.0177

57.	Feibus AH, Sartor O, Moparty K, et al. Clinical Use of PCA3 and TMPRSS2:ERG Urinary Biomarkers in African-American Men Undergoing Prostate Biopsy. *J Urol*. 2016;196(4):1053-1060. doi:10.1016/j.juro.2016.04.075

58.	MPS. LynxDx. Accessed September 17, 2022. https://www.lynxdx.com/my-prostate-score

59.	Ankerst DP, Goros M, Tomlins SA, et al. Incorporation of Urinary Prostate Cancer Antigen 3 and TMPRSS2:ERG into Prostate Cancer Prevention Trial Risk Calculator. *Eur Urol Focus*. 2019;5(1):54-61. doi:10.1016/j.euf.2018.01.010
