# Supplementary material for: Comparing diagnostic prostate cancer biomarkers using a framework of clinically meaningful criteria
Source: BJUI Compass. 2026 Sep 22;7(9):e70275. doi: 10.1002/bco2.70275 (PMC13595018; doi:10.1002/bco2.70275)
Supplement: Supplementary file 2 [file BCO2-7-e70275-s002.docx]

**SUPPLEMENTARY MATERIAL**

*Comparing Diagnostic Prostate Cancer Biomarkers Using a Framework of Clinically Meaningful Criteria*

**Supplementary Table 1. Systematic Literature Search Criteria**

| **Biomarker** | **Search Criteria** |
| --- | --- |
| *phi* | *((([−2]proenzyme) OR (p2PSA[Title/Abstract])) OR (prostate health index[Title/Abstract])) OR (phi[Title/Abstract])) AND (prostate cancer[Title/Abstract])* |
| *4KScore* | *(((((4k[Title/Abstract]) OR (4-kallikrein[Title/Abstract])) OR (four kallikrein[Title/Abstract])) OR (four-kallikrein[Title/Abstract])) OR (4kscore[Title/Abstract])) AND (prostate cancer)* |
| *SelectMDx* | *(((HOXC6[Title/Abstract]) AND (DLX1[Title/Abstract])) OR (SelectMDx[Title/Abstract])) AND (prostate cancer[Title/Abstract])* |
| *ExoDx* | *((ExoDx[Title/Abstract]) OR (EPI[Title/Abstract])) AND (prostate cancer[Title/Abstract])* |
| *MPS* | *((((((TMPRSS2:ERG[Title/Abstract]) AND (PCA3[Title/Abstract])) OR (MyProstateScore[Title/Abstract])) OR (MiPS[Title/Abstract])) OR (MPS[Title/Abstract])) OR (Mi-Prostate Score[Title/Abstract])) AND (prostate cancer[Title/Abstract])* |

PubMed, Scopus, and Web of Science were searched from January 2010 to September 2022 using the terms above for phi, 4KScore, SelectMDx, ExoDx, and MPS, following Preferred Reporting Items for a Systematic Review and Meta-analysis of Diagnostic Test Accuracy (PRISMA-DTA) methodology.
